# NIR Hyperspectral Imaging Technology Combined with Multivariate Methods to Study the Residues of Different Concentrations of Omethoate on Wheat Grain Surface

**DOI:** 10.3390/s19143147

**Published:** 2019-07-17

**Authors:** Liu Zhang, Zhenhong Rao, Haiyan Ji

**Affiliations:** 1 Key Laboratory of Modern Precision Agriculture System Integration Research, Ministry of Education, China Agricultural University, Beijing 100083, China; 2 Key Laboratory of Agricultural Information Acquisition Technology, Ministry of Agriculture, China Agricultural University, Beijing 100083, China; 3 College of Science, China Agricultural University, Beijing 100083, China

**Keywords:** hyperspectral imaging, omethoate, wheat kernels, multivariate scatter correction, neighborhood component analysis, support vector machine, object-wise

## Abstract

In this study, a hyperspectral imaging system of 866.4–1701.0 nm was selected and combined with multivariate methods to identify wheat kernels with different concentrations of omethoate on the surface. In order to obtain the optimal model combination, three preprocessing methods (standard normal variate (SNV), Savitzky–Golay first derivative (SG1), and multivariate scatter correction (MSC)), three feature extraction algorithms (successive projections algorithm (SPA), random frog (RF), and neighborhood component analysis (NCA)), and three classifier models (decision tree (DT), k-nearest neighbor (KNN), and support vector machine (SVM)) were applied to make a comparison. Firstly, based on the full wavelengths modeling analysis, it was found that the spectral data after MSC processing performed best in the three classifier models. Secondly, three feature extraction algorithms were used to extract the feature wavelength of MSC processed data and based on feature wavelengths modeling analysis. As a result, the MSC–NCA–SVM model performed best and was selected as the best model. Finally, in order to verify the reliability of the selected model, the hyperspectral image was substituted into the MSC–NCA–SVM model and the object-wise method was used to visualize the image classification. The overall classification accuracy of the four types of wheat kernels reached 98.75%, which indicates that the selected model is reliable.

## 1. Introduction

Wheat is one of the main food crops and is widely grown worldwide. Due to the long growth cycle of wheat, it generally undergoes several stages, such as emergence stage, tillering stage, jointing stage, heading stage, flowering stage, filling stage, and maturity stage. Wheat plants are most vulnerable to pests, such as aphids, during their growth and the most common method used by farmers is to spray the corresponding pesticides to prevent pests [1,2]. During the wheat growth cycle, it is generally required to spray pesticides. However, in order to pursue efficient insecticidal effects, some farmers usually use high-concentration pesticide solutions [3,4]. Moreover, most of the pesticides have strong adsorption properties. They are transported to the whole wheat plant through the spraying parts or residual pesticides in the soil, which will inevitably lead to pesticide residues in the wheat grains and endanger people’s health. Wheat kernels can be used to produce many products, such as bread, noodles, biscuits, and other foods. After fermentation, it can be used to make products such as beer, alcohol, and liquor (e.g., vodka). Fortunately, since there is a layer of bran on the surface of the wheat grain, a part of the pesticide can usually be removed after grinding into flour [5]. However, as people’s nutritional requirements are more balanced, wheat bran is also made into various foods [6]. For example, people use bran and flour to make whole wheat bread to achieve better dietary nutrition. Hence, it is important to use appropriate methods to detect wheat kernels containing pesticide residues.

Traditionally, thin layer chromatography, gas chromatography, and high performance liquid chromatography have been used to detect pesticide residues. Although these methods are highly accurate, they are complex, expensive, and time consuming, which is not conducive to large-scale promotion [7]. In recent years, with the development of non-destructive testing technology, some researchers often use technologies such as near-infrared spectroscopy and machine vision for rapid non-destructive testing. Machine vision often evaluates image features, such as sample color, shape, size, and surface texture characteristics, but cannot be applied to the analysis of chemical composition inside the sample [8]. Although near-infrared spectroscopy can acquire the spectral information of the sample, it acquires the spectral information of a single point based on the sample, and is easily affected by the conditions of sample uniformity and granularity, which will affect the accuracy of the established model [9]. Hyperspectral imaging technology is also a fast and non-destructive detection technology, which is widely used in agriculture and food. Because hyperspectral imaging technology integrates imaging technology and spectral analysis technology, it can not only acquire the spectral information of the sample but also the spatial information of the sample, thus overcoming the limitations of machine vision and near-infrared spectroscopy technology [10,11,12,13,14]. Many researchers have used hyperspectral imaging techniques to conduct a series of studies on pesticide residues. Ren et al. [15] used hyperspectral imaging technology to identify the residues of different concentrations of omethoate on the surface of spinach, using the chi-square test and fusion with various machine learning algorithms to finally realize the differentiation of different concentrations of omethoate spinach on the surface. Shao et al. [16] used near-infrared hyperspectral imaging technology and established partial least squares discriminant analysis combined with a competitive adaptive reweighted sampling algorithm (CARS–PLSDA), partial least squares discriminant analysis combined with full wavelengths (FW–PLSDA), and linear discrimination analysis combined with regression coefficients (RC–LDA) models to distinguish the pesticide types on the surface of chlorella. Finally, the classification accuracy of the RC–LDA model reached 97% and was selected as the best model. Li et al. [17] successfully used the image information of hyperspectral imaging technology combined with principal component analysis to detect pesticide residues on the surface of “Gannan navel” orange. The above results indicate the feasibility of using hyperspectral imaging techniques to detect pesticide residues.

To the best of our knowledge, few people have used hyperspectral imaging to detect pesticide residues in cereal crops such as wheat kernels. Hence, this study mainly explores the feasibility of using hyperspectral imaging technology combined with multivariate methods to detect different concentrations of omethoate residues on wheat grain surface. The specific objectives are as follows: (1) Using different spectral pretreatment methods to preprocess the original spectral data, then combining with different classifier models for full-band wavelength modeling analysis, and selecting the best spectral preprocessing method; (2) utilizing different feature extraction algorithms to extract the characteristic wavelengths of the spectral data processed by the optimal preprocessing method, then combining with different classifier models for feature wavelength modeling analysis, and selecting the best model; (3) substituting the hyperspectral image into the best model, visualizing the classification effect of the four types of wheat grains, so as to verify the reliability of the selected model.

## 2. Materials and Methods

### 2.1. Sample

The pesticide used in the experiment was 40% omethoate (molecular formula: C_5_H_12_NO_4_PS) emulsifiable concentrate, which was often used to prevent wheat aphids and other spur-type pests. These wheat grain samples were provided by Wudeli Flour Co., Ltd. (Zhengzhou, China), and they were hand-screened by experienced professional inspectors in the quality inspection department to ensure that they were healthy wheat grains. The omethoate emulsifiable concentrate was prepared into a solution of different concentration gradients in a ratio of 1:100, 1:500, and 1:1000 with distilled water (in which 1:500 and 1:1000 are the pesticide concentration ratios commonly used by farmers). A total of 400 wheat kernels of similar size were selected from these healthy wheat grains, and divided into 4 groups (one of which was not treated with pesticide dropping), each group consisting of 100 grains, and all of them were arranged with the back facing up. Subsequently, different concentrations of the pesticide solution were added to the back of the wheat grain by a pipette, and 10 μL per kernel. In order to ensure that the moisture on the surface of the wheat grain was lost as much as possible to reduce the interference in subsequent experiments, the wheat grains were placed in a dry, ventilated, and room-temperature environment at 20 °C for one week after the end of the pesticide dropping experiment. Then, we performed hyperspectral image acquisition.

### 2.2. Hyperspectral Imaging System

**Figure 1:**
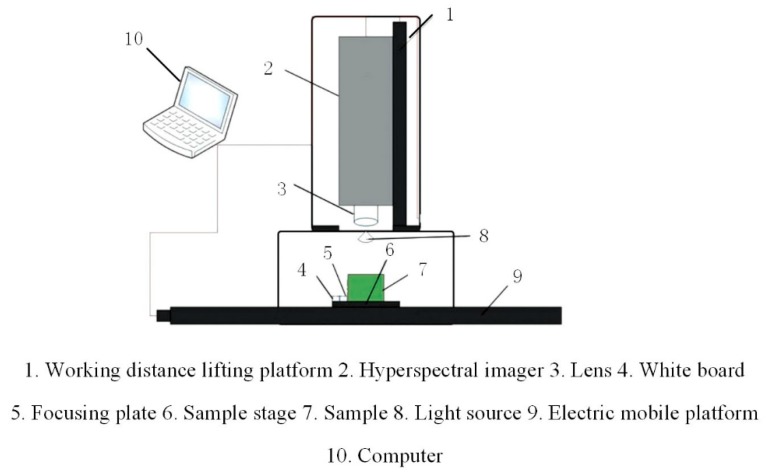
Structure diagram of hyperspectral imaging system.

The experiment selected the “GaiaSorter” hyperspectral imaging system produced by Beijing Zolix Co., Ltd. (Beijing, China). The core components of the system consist of uniform light source, spectral camera, computer, and related control software. The camera used in the spectral imager is Zolix’s “image-λ” series hyperspectral camera with a spectral range of approximately 866.4–1701.0 nm. The uniform light source consists of four 200 W bromine tungsten, as shown in Figure 1. The working principle of the system is to place the sample to be tested on the electric mobile platform that is controlled by software, and collect the sample image according to the push-broom method [18]. With the movement of the electric moving platform, the hyperspectral cube data containing the spectral information and image information of the tested sample are finally obtained.

### 2.3. Hyperspectral Image Data Acquisition and Correction

Before the data acquisition, the power supply was turned on to warm up the hyperspectral imaging system for 30 min to eliminate errors, such as baseline drift caused by the system. Then the system’s own SpecVIEW software (SpecView Ltd., Uckfield, UK) was run, performing a series of tests such as focusing, and finally, we determined the exposure time as 0.09 s, and the moving speed of the electric mobile platform as 0.55 cm/s. In order to avoid external light interference during the data acquisition, the system’s special glass baffle was used to block the window around the system. Finally, four hyperspectral images were collected, each with 100 wheat grains. After the data were collected, the original hyperspectral image needed to be corrected in black and white to reduce the influence of dark current and noise on the image [19]. The specific operation steps are as follows: In the same collection environment, the image captured by the camera at the white board is W, then the camera lens cover is closed, the light is turned off, and the black image collected is B. The correction formula is:

(1) $$R=\frac{I\;-\;B}{W\;-\;B}$$

In Formula (1), R is the corrected hyperspectral image, I is the original spectral image, B is the all-black calibration image, and W is the all-white calibration image. The software tool for correcting images is SpecVIEW (the control software of “GaiaSorter” Hyperspectral imaging system). Subsequent software used is ENVI5.3 (ITT Visual Information Solutions, Boulder, UT, USA) and Matlab2018b (The Math Works, Natick, MA, USA).

### 2.4. Data Analysis

#### 2.4.1. Spectral Data Extraction and Preprocessing

**Figure 2:**
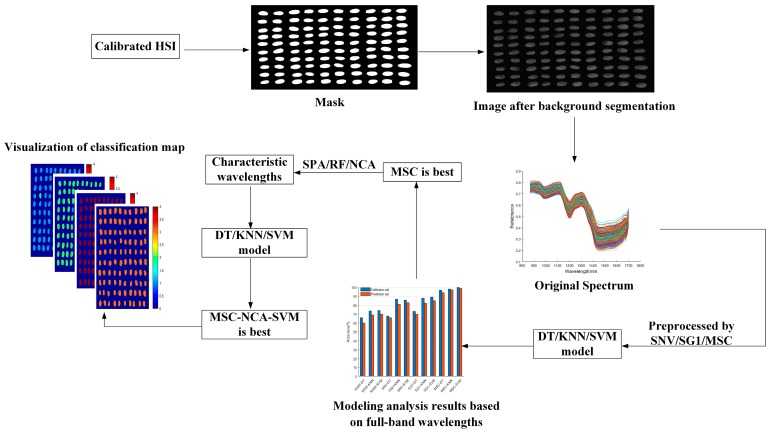
Main steps of extracting spectral information, characteristic wavelength, and establishing discrimination model.

Due to the complexity of the hyperspectral cube data acquired by the hyperspectral imaging system, it is necessary to perform a series of processing on the original image to finally extract the spectral data, as shown in Figure 2: (1) Using the Sobel operator to extract the edge of the wheat grain in the corrected hyperspectral image, expand and erode; (2) Binary processing generates a mask, and the original image is segmented to remove the background; (3) Extracting the entire area of each wheat grain on the image as the region of interest (ROI), and then calculating the average reflectance of all the pixels in the selected area as the spectral value of each wheat grain. During this time, image segmentation was performed on Matlab2018b software, and the average spectral value of all pixels in the ROI was calculated on ENVI 5.3 software.

Since noise may be introduced due to some interference factors during data acquisition, it is necessary to preprocess the original spectral data to improve the accuracy of modeling. In this experiment, three spectral pretreatment methods (SNV, SG1, and MSC) were used to preprocess the original spectral data, respectively.

#### 2.4.2. Characteristic Wavelength Extraction

Since the full-band spectral information and spatial information of the original hyperspectral data are redundant and collinear, this interference information will affect the robustness, accuracy, and calculation speed of the model in the subsequent modeling process. Therefore, the extraction of characteristic wavelengths by appropriate methods is critical for spectral analysis, and these characteristic wavelengths are very useful for developing multispectral online detection systems. In this study, the successive projections algorithm (SPA), random frog (RF), and neighborhood component analysis (NCA) are used to extract the characteristic wavelengths, respectively.

SPA is a vector-variable, collinearity-minimizing, forward variable selection algorithm. Its advantage lies in extracting several characteristic wavelengths of the whole band, which can eliminate the redundant information in the original spectral matrix and can be used for the screening of spectral characteristic wavelengths [20].

The RF algorithm is a variable selection method, which is similar to the reversible jump Markov chain Monte Carlo (RJMCMC) algorithm [21]. It calculates the probability of selection for each variable by simulating a Markov chain obeying a steady-state distribution in the model space, so as to evaluate the importance of variables. The main operational steps of the random frog algorithm are as follows: (1) Initialization parameters, randomly generating a subset of initial variables V_0_ containing Q variables; (2) generating a random number according to the standard normal distribution, and taking the nearest integer Q* from the random number as the number of variables included in the candidate variable subset V*; (3) according to the relationship between Q* and Q, establishing a subset of candidate variables V* containing Q* variables; (4) determining V* according to the probability of root mean square error of cross-validation (RMSCEV) calculation, and letting V_1_ = V* until N iterations terminate the process; (5) Calculating the selection probability of each variable, and using this as the criterion for selecting variables.

NCA is a non-parametric and embedded method for selecting features, whose purpose is to maximize the prediction accuracy of regression and classification. The principle of NCA is based on the K-nearest neighbor (KNN) measured by the Mahalanobis distance. By continuously optimizing the accuracy of the KNN classification, the transformation matrix is learned, and finally, the transformation matrix for reducing the original data is obtained [22]. Its main steps are as follows: (1) Consider a random classifier and randomly select a point *Ref*(X) from the data set S = {(X***_i_***,Y***_i_***), *i* = 1, 2, ..., *n*} (where X***_i_*** is the input sample and Y***_i_*** is the label corresponding to the sample) as the reference point of X, which is similar to the 1-NN classifier; (2) the probability P(*Ref*(X*_i_*) = X*_j_*|S) indicates that the point X*_j_* selected from S is the point closest to the distance X***_i_*** and is determined by the distance function; (3) calculate the weight vector *w* and determine the average probability F(*w*) of the correct classification of the random classifier (the goal of the NCA is to maximize F(*w*)); (4) the objective function is optimized by the gradient descent method to find the most suitable transformation matrix.

#### 2.4.3. Discriminant Model

The Decision Tree (DT) is a very common classification method. It determines the probability that the expected value of net present value is greater than or equal to zero by constructing a decision tree according to the probability of occurrence in various situations. The decision tree in machine learning represents a mapping relationship between object attributes and object values [23]. Each node in the tree represents an object, and each forked path represents a possible attribute value, and each leaf node corresponds to the value of the object represented by the path from the root node to the leaf node. Since the decision tree is a graphical method using probability analysis, it is very intuitive. This study used cross-validation to determine the most appropriate minimum leaflet (minleaf) value.

The core idea of the K-nearest neighbor (KNN) algorithm is that it can be classified or regressed based on the distance between different eigenvalues. It determines the category to which the entire sample belongs based on the category of the nearest one or several samples [24]. The KNN is both a theoretically mature algorithm and one of the simplest algorithms in machine learning. This study used cross-validation to determine the K value of the model.

The support vector machine (SVM) is based on structural risk minimization, finds a segmentation hyperplane between the data, maximizes the spacing, and achieves data classification. SVM generally has linear kernel function, polynomial kernel function, radial basis function (RBF) kernel function, etc. Different kernel functions have different functions [25]. This study uses the RBF kernel function, which has the advantage of mapping the characteristics of the data to an infinite dimension and making it easy to select parameters. Finally, the grid search procedure is used to determine the penalty coefficient (c) and the kernel range (g).

## 3. Results and Discussion

### 3.1. Original Spectral Analysis

**Figure 3:**
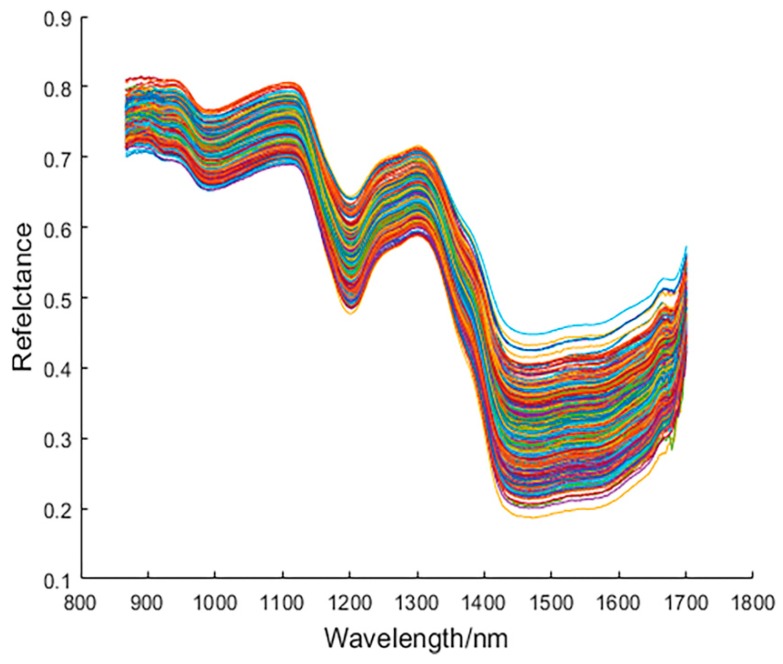
Original spectral curves of all samples.

**Figure 4:**
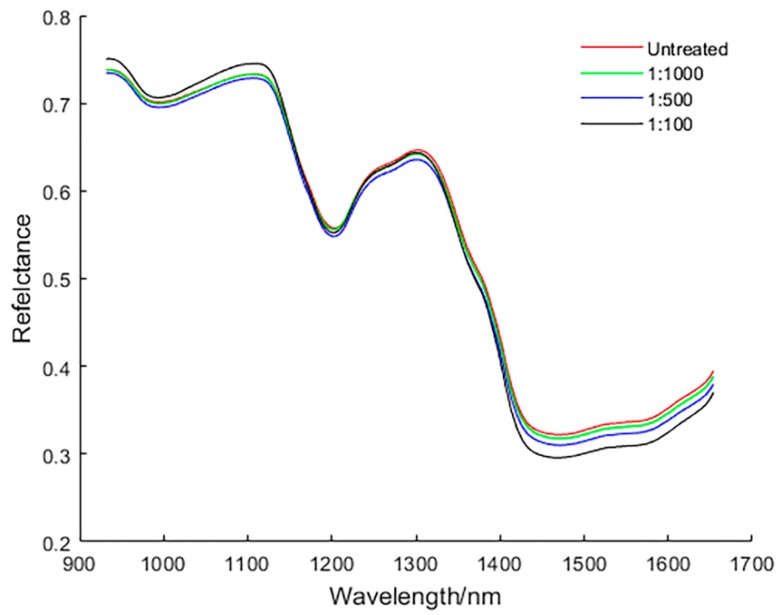
Average spectral curves of wheat grains with different concentrations of omethoate.

Figure 3 shows the original spectral curves of all samples with a wavelength range of 866.4–1701.0 nm for a total of 254 wavelengths. There is obvious noise in the front and rear part of the original spectral curve, due to the influence of optical instruments and other factors. Therefore, only the spectra of 931.8–1653.8 nm were selected and a total of 220 wavelengths were used for analyses. Figure 4 shows the average spectral curves of wheat grains with different concentrations of pesticide residues and healthy wheat grains in the range of 931.8–1653.8 nm. It can be seen from Figure 4 that the spectral trends of the four types of wheat kernels are very similar and are not well distinguished; however, in the range of 1400– 1650 nm, the difference in reflectance of the four types of wheat grains is the most obvious, and the order of spectral reflectance values from high to low is: Untreated > 1:1000 > 1:500 > 1:100. This is due to the second overtone of combination C–H and the second overtone of N–H in the range of 1400–1650 nm [26], which is related to the absorption of omethoate. Overall, the average spectral curve of wheat grain with a 1:100 concentration of pesticides differed most from healthy wheat grain (no pesticides); however, the difference between the average spectral curve of healthy wheat grain and wheat grain with a 1:500 and 1:1000 concentration of pesticide is very small.

### 3.2. Modeling Analysis Based on Full Wavelengths

All samples were randomly divided into a calibration set and a prediction set in a 3:1 ratio prior to modeling. Healthy wheat kernels and wheat kernels with 1:1000, 1:500, and 1:100 concentrations of omethoate were numbered 1, 2, 3, and 4 as their respective labels (Y variable). The calibration set contains a total of 300 wheat kernels (i.e., “1” has 77 kernels, “2” has 69 kernels, “3” has 75 kernels, and “4” has 79 kernels), and the prediction set contains 100 wheat kernels (i.e., “1” has 23 kernels, “2” has 31 kernels, “3” has 25 kernels, and “4” has 21 kernels).

**Figure 5:**
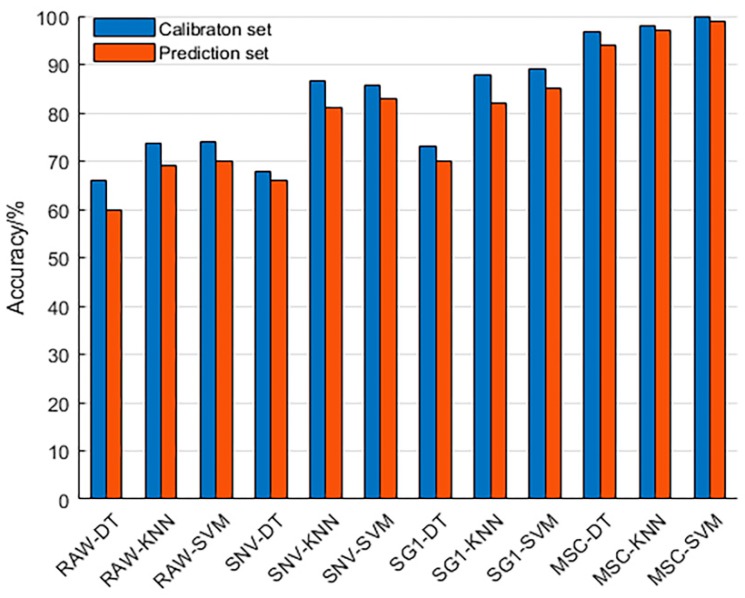
Modeling analysis results based on full wavelengths.

In order to select the best combination of pretreatment method and model, the original spectral data and the spectral data preprocessed by SNV, SG1, and MSC were respectively input into DT, KNN, and SVM classifier models for comparison with each other. Figure 5 shows the results of a calibration set and a prediction set based on various preprocessing methods of full wavelengths combined with different classifier models, where Raw represents raw spectral data. It can be seen from Figure 5 that the modeling accuracy of the spectral data after processing with MSC is the highest. The accuracy of the calibration set and prediction set of the MSC–DT, MSC–KNN, and MSC–SVM models are both higher than 90%, which is significantly better than the modeling results of other preprocessing methods. This may be due to differences in the sample itself and other factors, such as optical instruments in this experiment, which cause scattering of light to introduce noise. MSC can largely eliminate the effects of light scattering and spectral baseline drift [27].

In order to improve the calculation speed and robustness of the model, it is necessary to reduce the dimension of the spectral data. Finally, the spectral data processed by the MSC are selected for feature wavelength extraction, and further modeling analysis is performed to determine the final model combination.

### 3.3. Characteristic Wavelength Extraction Based on SPA, RF, and NCA

Due to the high dimensionality of the hyperspectral cube data, the collinearity of the information between the bands leads to information redundancy. It affects the convergence of the model and is also very disadvantageous for the subsequent development of multispectral online detection systems. Therefore, selecting a smaller number and a very useful characteristic band is very advantageous for improving the operation speed and stability of the model. In this study, the SPA, RF, and NCA were used to extract the characteristic wavelength from 220 variables, respectively.

**Figure 6:**
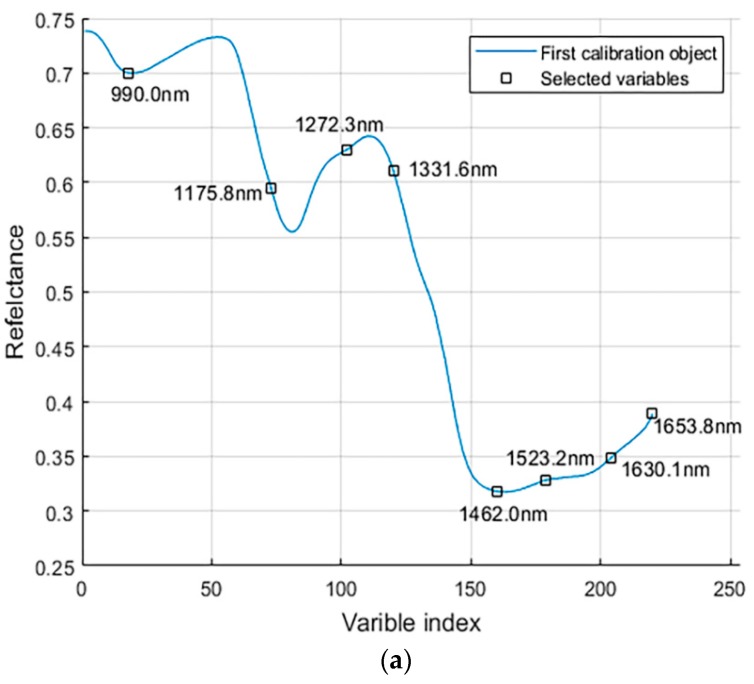

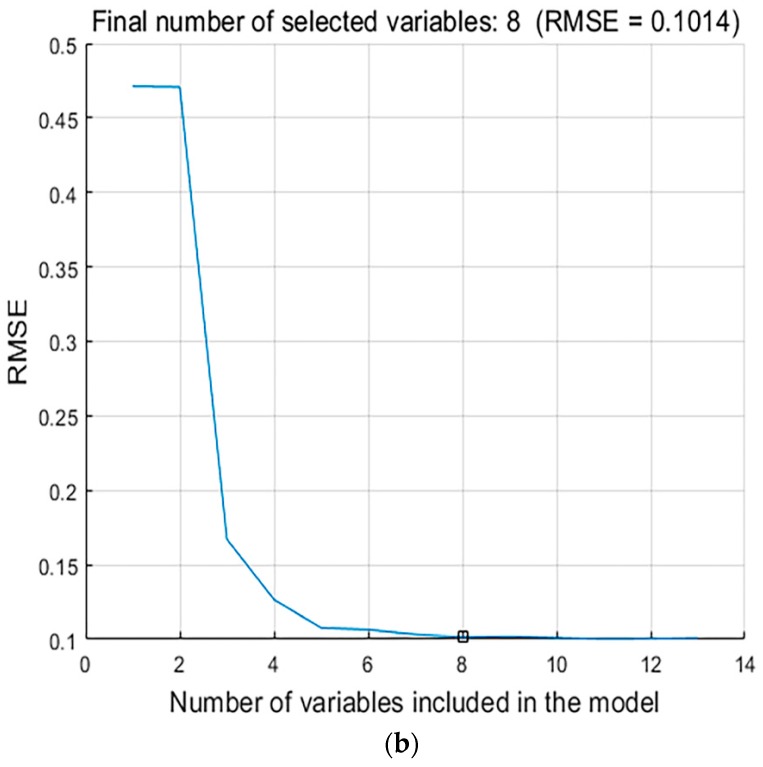
Process of selecting characteristic wavelengths based on successive projections algorithm (SPA). (**a**) Final number of selected variables according to the minimum root mean square error of validation (RMSEV) in the validation set of multiple linear regression (MLR); (**b**) the selected characteristic wavelength marked with a black square.

In the SPA, the characteristic wavelength was determined based on the minimum root mean square error of validation (RMSEV) in the validation set of multiple linear regression (MLR) calibration. Finally, eight characteristic wavelengths (990.0, 1175.8, 1272.3, 1331.6, 1462.0, 1523.2, 1603.1, and 1653.8 nm) were extracted, as shown in Figure 6a,b. In addition, the characteristic wavelength based on SPA extraction is reduced by more than 96% ($\frac{220-8}{220}=96.36\%$) with respect to the full wavelength data, which obviously can improve the calculation speed of the model. The 990.0 nm is related to the third overtone vibration of N–H. The 1175.8 nm mainly corresponds to the third overtone of C–H. The 1272.2, 1331.6, and 1523.2 nm correspond to the second overtone of the C–H combination. The 1462.0 nm is associated with the third overtone stretching of C=O. The vicinity of 1650 nm is related to the first overtone of C–H [28].

**Figure 7:**
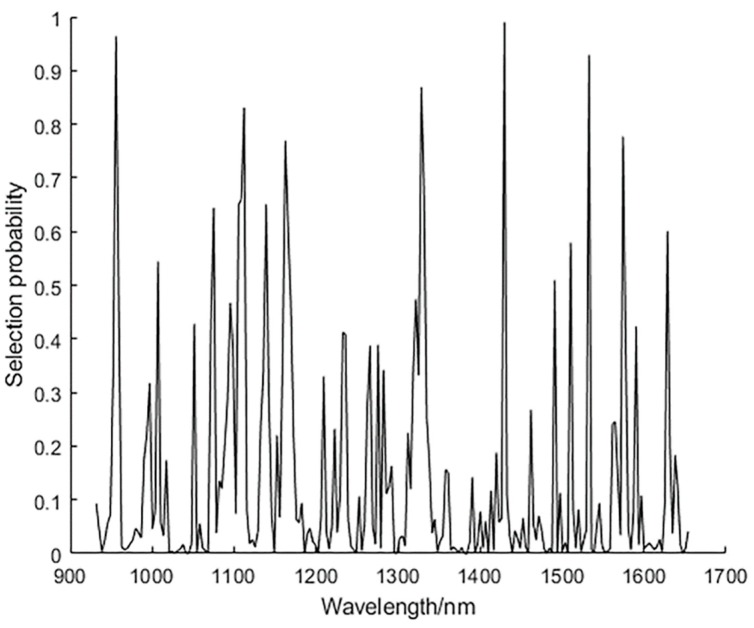
Using random frog (RF) algorithm to show the selection probability of each wavelength.

In this experiment, the number of operations N of the RF algorithm is set to 1000, the number of potential variable A is 10, and the number of variable Q in the initial model of the leapfrog is 2. After the operation, the selection possibility corresponding to each variable index is used as a screening basis, and the selection possibility size is arranged in descending order. Figure 7 shows the result of the RF algorithm extracting feature wavelengths, where each wavelength variable corresponds to a variable address. By selecting the threshold of 0.7, seven characteristic wavelengths were finally selected, which were 955.8, 1121.1, 1162.4, 1328.3, 1429.6, 1532.8, and 1574.4 nm, respectively. The 955.8 nm is related to the third overtone vibration of N–H. The 1121.1 and 1162.4 nm mainly correspond to the third overtone of C–H. The 1328.3 and 1532.8 nm correspond to the second overtone of the C–H combination. The 1429.6 nm is associated with the third overtone stretching of C=O. The 1574.4 nm corresponds to the second overtone of N–H [28].

**Figure 8:**
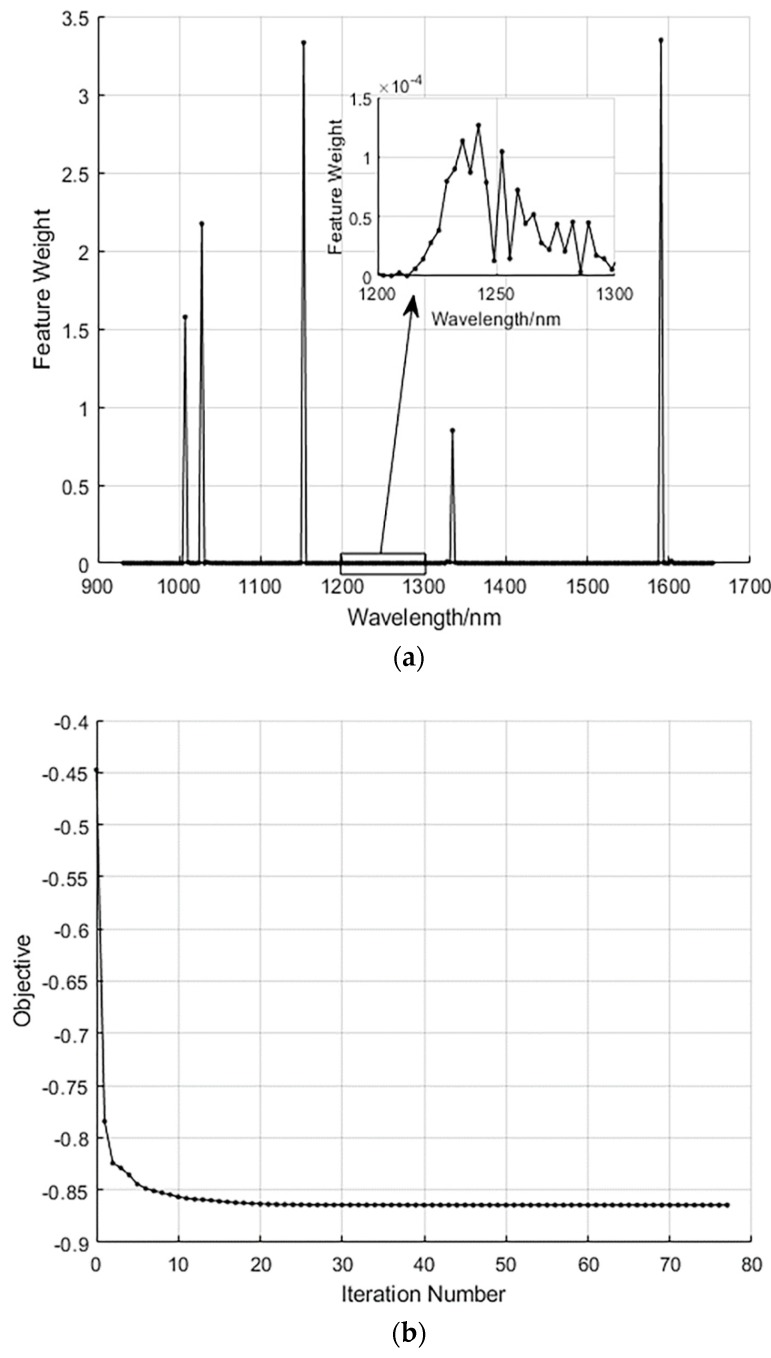
Process of selecting characteristic wavelengths based on neighborhood component analysis (NCA). (**a**) Weight value for each wavelength; (**b**) the relationship between the objective function value and the number of iterations.

The NCA algorithm is often applied to the selection of feature variables in high-dimensional data. It can calculate the weight value of each variable in the operation process, and the weight value of the irrelevant or low correlation variable is assigned 0 or very close to 0, after which a variable with a high weight value can be selected as the feature value. Figure 8a,b shows the process of extracting characteristic wavelengths by the NCA algorithm. Figure 8a shows that only 6 of the 220 wavelengths have a weight value significantly higher than 0, while other wavelengths have a weight value of 0 or very close to 0. Figure 8b shows the process of optimizing the objective function value with the number of iterations. When the number of iterations is 20, the value of the objective function tends to be stable, indicating that the final selected transform matrix is suitable. Therefore, a total of six characteristic wavelengths were selected, which were 1007.1, 1027.5, 1152.4, 1334.9, and 1590.4 nm, respectively. The 1007.1 and 1027.5 nm are related to the third overtone vibration of N–H. The 1152.1 nm mainly corresponds to the third overtone of C–H. The 1334.9 nm corresponds to the second overtone of the C–H combination. The 1590.4 nm corresponds to the second overtone of the N–H [28].

Table 1 shows the characteristic wavelengths extracted by three feature extraction algorithms. In summary, the characteristic wavelength extracted by the SPA algorithm is close to the characteristic wavelength extracted by the RF algorithm, and the number of characteristic wavelengths extracted by the NCA is the least. Since omethoate contains a large amount of C–H, as well as some C=O and N–H, these characteristic wavelengths extracted with SPA, RF, and NCA have a great correlation with omethoate.

### 3.4. Modeling Analysis Based on Characteristic Wavelength

In this section, all samples participating in the calibration set and the prediction set of wheat kernels are identical to the full spectrum band-based modeling analysis. In order to further select the best model combination, this study combines the spectral data of MSC processing with different feature selection methods, and inputs three kinds of classifier models to finally select the best model combination. Table 2 shows the results of modeling analysis of different feature extraction algorithms combined with DT, KNN, and SVM models. In general, the results based on characteristic wavelength modeling are basically the same as the accuracy of modeling results based on full wavelengths, and the accuracy of the calibration set and prediction set of each model is higher than 90%. This shows that the characteristic wavelengths extracted by these feature extraction algorithms is useful. Specifically, in the modeling analysis of three different feature extraction algorithms (SPA, RF, and NCA) combined with three classifier models (DT, KNN, and SVM), the calibration set and prediction set of the DT model have the lowest accuracy, while the SVM model works best. The characteristic wavelengths extracted using the NCA algorithm relative to the SPA and RF algorithms perform best in the three classifier models.

In summary, the accuracy of the calibration set and prediction set of the MSC–NCA–SVM model is the highest, at 99.67% and 99%, respectively. Therefore, it is selected as the best model.

### 3.5. Visualization of Wheat Grain with Different Concentrations of Pesticides

In order to verify the reliability of the selected model, it is necessary to classify the pixels of the hyperspectral image and visualize the classification effect. In the past, many researchers used object-wise and pixel-wise methods to visualize classification effects. The object-wise method is to use the average spectral data of all pixels as the spectral data for each kernel, while the pixel-wise method predicts the categories of all pixels in a kernel based on the spectral data of the single-point pixel.

Baek et al. [29] used the established PLS–DA model to visualize viable soybean seeds and non-viable soybean seeds based on the pixel-wise method (by setting a pixel threshold and calculating the percentage of all the pixel values above the threshold on the whole kernel as the classification basis) and object-wise method, respectively, and it turns out that the object-wise method achieves better classification results. In practical production, pesticides may be unevenly distributed on the surface of wheat grains (i.e., some wheat grains have fewer pesticides on the surface). If the pixel-wise method is used, some wheat grains containing pesticides may be classified into wheat grains without pesticides, and the object-wise method can avoid this drawback. Therefore, this study used the object-wise method to visualize wheat grains containing different concentrations of pesticides.

**Figure 9:**
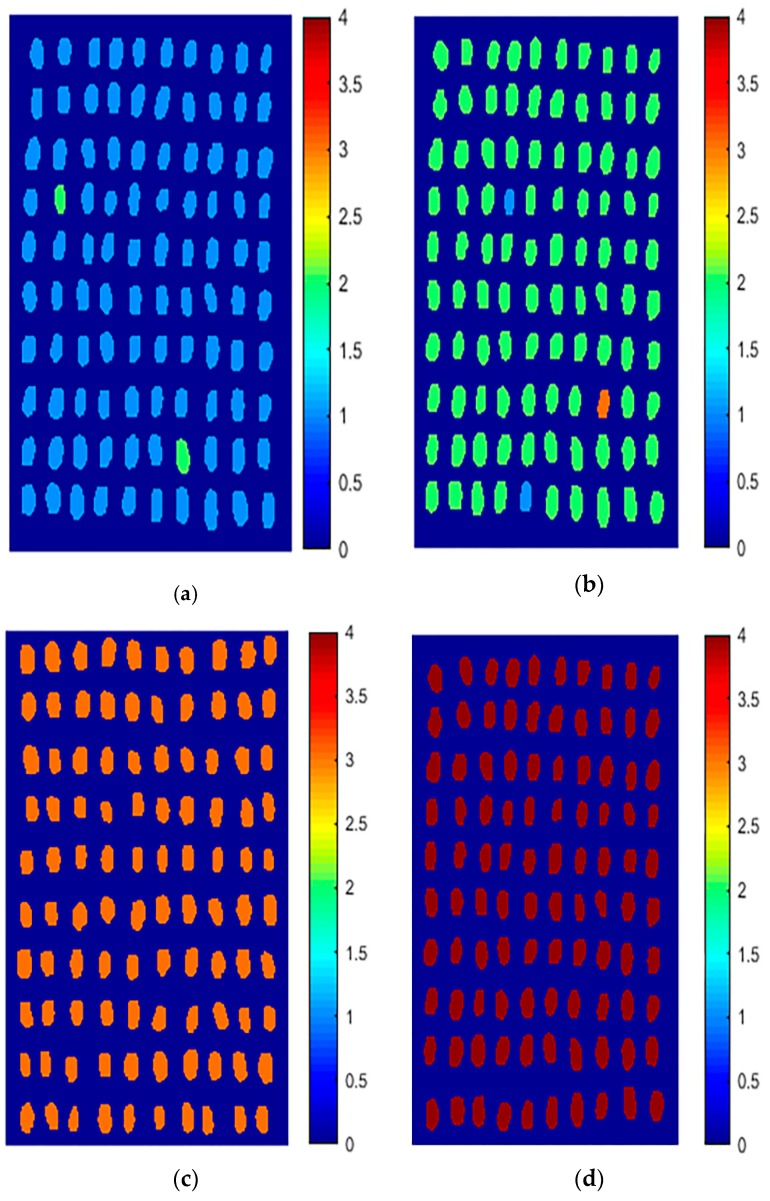
Visualization classification of wheat seeds with different concentrations of omethoate. (**a**) Healthy wheat kernels; (**b**) 1:1000; (**c**) 1:500; (**d**) 1:100 (Nos. 1, 2, 3, and 4 representing healthy wheat kernels, wheat kernels containing 1:1000 concentration of omethoate, wheat kernels containing 1:500 concentration of omethoate, and wheat kernels containing 1:100 concentration of omethoate, respectively).

The hyperspectral image of the sample was substituted into the MSC–NCA–SVM model and classified using an object-wise approach. Figure 9a–d are images of visualization of four types of wheat kernels, and the color of each type of wheat grain in Figure 9 corresponds to the value in the chroma bar. It can be seen that the visualized image classification effect of four categories of wheat grains is very good, and the overall classification accuracy is 98.75% (395/400), indicating the reliability of the selected model.

## 4. Conclusions

In this study, hyperspectral cube data with different concentrations of pesticide wheat grains were obtained by selecting the hyperspectral imaging system of 866.4–1701.0 nm. The model was established by a multivariate method and the image classification results were visualized to verify the reliability of the selected model. The conclusions are as follows:

The original spectral data were preprocessed by SNV, SG1, and MSC respectively, and then combined with DT, KNN, and SVM classifiers for full wavelengths modeling analysis. It was found that the spectral data of MSC pretreatment performed best in three classifiers (the accuracy of the calibration set and the prediction set were both higher than 90%). In order to reduce the dimension of the original spectrum and improve the performance of the model, the MSC preprocessed spectral data were selected for feature wavelength extraction.

(1) The spectral data of the MSC processed data were extracted by SPA, RF, and NCA, respectively, and then the characteristic wavelengths were modeled and analyzed by combining DT, KNN, and SVM classifier models. By comparison, it was found that the modeling results based on characteristic wavelengths were very close to the results based on full-wavelength modeling, which indicates the reliability of the characteristic wavelengths extracted by the three feature algorithms. Further analysis shows that the NCA algorithm performs best in the three feature extraction algorithms, and the SVM performs best in the three classifier models. Therefore, the MSC–NCA–SVM model is finally selected as the best model;
(2) In order to verify the reliability of the selected model, the hyperspectral image was substituted into the MSC–NCA–SVM model, and the classification effect was visualized using the object-wise method. The results showed that only 5 of the 400 wheat kernels were misclassified, and the overall classification accuracy was 98.75%, indicating the reliability of the selected model.

The above results indicate the feasibility of using hyperspectral imaging technology combined with multivariate data analysis to identify wheat seeds with different concentrations of pesticides, which can provide a reference for the development of multi-spectral online detection systems in the future. However, there may be more than one species of pesticide remaining on the surface of wheat grains in actual production. Therefore, in subsequent research, different pesticide types and more pesticide solution gradients will be tested according to the actual situation, which will provide a greater basis for the future development of multi-spectral online detection systems.

## Figures and Tables

**Table 1:** Characteristic wavelength selected based on SPA, RF, NCA.

Methods	NO.	Characteristic Wavelengths (nm)
SPA	8	990.0, 1175.8, 1272.3, 1131.6, 1462.0, 1523.2, 1603.1, 1653.8
RF	7	995.8, 1121.1, 1162.4, 1328.3, 1429.6, 1532.8, 1574.4
NCA	5	1007.1, 1027.5, 1152.4, 1334.9, 1590.4

**Table 2:** Modeling analysis results based on characteristic wavelengths.

Model	Parameter	Accuracy of Calibration (%)	Accuracy of Prediction (%)				
		Ovreall	1	2	3	4	Overall
MSC–SPA–DT	30	95.67	95.65	90.32	92	95.24	93
MSC–SPA–KNN	9	97.67	100	93.55	92	100	96
MSC–SPA–SVM	(2, 0.125)	99.33	100	96.77	96	100	98
MSC–RF–DT	10	99.67	95.65	90.32	96	95.24	94
MSC–RF–KNN	5	98	91.30	93.55	96	100	96
MSC–RF–SVM	(2, 0.25)	99	95.65	100	96	100	98
MSC–NCA–DT	8	96.33	95.65	90.32	100	95.24	94
MSC–NCA–KNN	7	98	95.65	90.32	100	100	97
MSC–NCA–SVM	(2, 0.25)	99.67	100	96.77	100	100	99

Parameter for DT is “minleaf” value, parameter for KNN is number of neighbors (k) and parameters for SVM are c and g.
